# Genetic Mapping by Integration of 55K SNP Array and KASP Markers Reveals Candidate Genes for Important Agronomic Traits in Hexaploid Wheat

**DOI:** 10.3389/fpls.2021.628478

**Published:** 2021-02-23

**Authors:** Hongchun Xiong, Yuting Li, Huijun Guo, Yongdun Xie, Linshu Zhao, Jiayu Gu, Shirong Zhao, Yuping Ding, Luxiang Liu

**Affiliations:** National Engineering Laboratory for Crop Molecular Breeding, National Center of Space Mutagenesis for Crop Improvement, Institute of Crop Sciences, Chinese Academy of Agricultural Sciences, Beijing, China

**Keywords:** QTL, heading date, plant height, thousand grain weight, spike length, wheat

## Abstract

Agronomic traits such as heading date (HD), plant height (PH), thousand grain weight (TGW), and spike length (SL) are important factors affecting wheat yield. In this study, we constructed a high-density genetic linkage map using the Wheat55K SNP Array to map quantitative trait loci (QTLs) for these traits in 207 recombinant inbred lines (RILs). A total of 37 QTLs were identified, including 9 QTLs for HD, 7 QTLs for PH, 12 QTLs for TGW, and 9 QTLs for SL, which explained 3.0–48.8% of the phenotypic variation. Kompetitive Allele Specific PCR (KASP) markers were developed based on sequencing data and used for validation of the stably detected QTLs on chromosomes 3A, 4B and 6A using 400 RILs. A QTL cluster on chromosome 4B for PH and TGW was delimited to a 0.8 Mb physical interval explaining 12.2–22.8% of the phenotypic variation. Gene annotations and analyses of SNP effects suggested that a gene encoding protein Photosynthesis Affected Mutant 68, which is essential for photosystem II assembly, is a candidate gene affecting PH and TGW. In addition, the QTL for HD on chromosome 3A was narrowed down to a 2.5 Mb interval, and a gene encoding an R3H domain-containing protein was speculated to be the causal gene influencing HD. The linked KASP markers developed in this study will be useful for marker-assisted selection in wheat breeding, and the candidate genes provide new insight into genetic study for those traits in wheat.

## Introduction

Wheat (*Triticum aestivum* L.) is one of the most important cereal crops worldwide, providing a food source for 30% of the human population (Mayer et al., 2014). Improving the yield potential of wheat is of great significance for meeting the food demand from an increasing population (Tshikunde et al., 2019). Agronomic traits such as heading date (HD), plant height (PH), thousand grain weight (TGW), and spike length (SL) are important factors affecting yield and always targeted by wheat breeders (Tshikunde et al., 2019). Recent advances in wheat genomics have accelerated the genetic dissection of important agronomic traits, and a large number of quantitative trait loci (QTLs) for these traits have been identified (Rasheed and Xia, 2019).

Heading date is crucial for adaptation to different environments and yield stability in wheat (Snape et al., 2001). Over a hundred QTLs for HD located across all wheat chromosomes have been detected (Milec et al., 2014; Kiseleva and Salina, 2018). The cloned genes affecting HD or flowering in wheat are mainly classified into three groups: vernalization (*VRN*), photoperiod (*Ppd*), and earliness *per se* (*Eps*) genes (Snape et al., 2001). Four *VRN* genes (*VRN1*, *VRN2*, *VRN3*, and *VRN4*) located on chromosome 5 or 7 of the A/B/D genomes, have been identified by map-based cloning (Yan et al., 2003, 2004, 2006; Kippes et al., 2015; Xie et al., 2019). The *Ppd* genes for photoperiod responses in wheat are mainly located on chromosomes 2A, 2B, and 2D (Beales et al., 2007). The *Eps* genes were identified on chromosome 1A^*m*^ in *Triticum monococcum* (Alvarez et al., 2016) and on long arm of chromosome 1D in hexaploid wheat (Zikhali et al., 2014).

Plant height is another important factor affecting yield potential in wheat (Flintham et al., 1997). Twenty-five reduced height genes (*Rht*), *Rht1* to *Rht25*, have been identified in wheat (Mo et al., 2018). According to the distinct responses to exogenous gibberellic acid (GA), these *Rht* genes were classified into GA-sensitive or GA-insensitive categories (Lou et al., 2016). The “green revolution” genes *Rht-B1b* (*Rht1*) and *Rht-D1b* (*Rht2*) located on chromosome 4B and 4D, respectively, encode truncated DELLA proteins, which are involved in the gibberellin signaling pathway (Peng et al., 1999). *Rht4*, *Rht5*, *Rht7*, *Rht8*, *Rht9*, *Rht12*, *Rht13*, *Rht22*, and *Rht23* are located on 2B, 3B, 2A, 2D, 7B, 5A, 7B, 7A, and 5D, respectively (Peng et al., 1999; Ellis et al., 2005; Asplund et al., 2012; Chen et al., 2015; Vikhe et al., 2017). *Rht24* is located on 6AL (Tian et al., 2017; Wurschum et al., 2017) while *Rht14*, *Rht16*, *Rht18, and Rht25* are located on 6AS (Haque et al., 2011; Grant et al., 2018; Mo et al., 2018).

TGW is one of the three essential components of grain yield. Most of the cloned genes associated with TGW in wheat were identified using a homology-based strategy (Chen et al., 2020). The wheat *TaGL3-5A* gene has been cloned, and a SNP in the 11^*th*^ exon of *TaGL3-5A* is associated with variation in grain length and TGW (Yang et al., 2019). In addition, the *TaGW2* gene in wheat is well studied for its function in regulating grain weight (Su et al., 2011; Bednarek et al., 2012; Jaiswal et al., 2015; Simmonds et al., 2016; Zhai et al., 2018; Zhang et al., 2018). Through genetic linkage analyses, stable QTLs explaining over 10% of the phenotypic variance for TGW were identified on chromosomes 1A (Varshney et al., 2000), 1B (Mir et al., 2012), 2D (Ma et al., 2019), 3A (Cui et al., 2014a), 3D (Cui et al., 2014a; Kumar et al., 2016), 4A (Araki et al., 1999), 4B (Kumar et al., 2016; Guan et al., 2018; Xu et al., 2019; Chen et al., 2020), 5A (Börner et al., 2002; Cuthbert et al., 2008; Mir et al., 2012; Kumar et al., 2016), 5B (Yang et al., 2020), 5D (Li et al., 2018), 6A (Mir et al., 2012), 6D (Cui et al., 2014a), 7A (Kumar et al., 2006, 2016; Mir et al., 2012), and 7D (Chen et al., 2020).

Spike architecture traits such as spike length (SL) are tightly related to grain production in wheat (Yao et al., 2019). A number of studies have identified stable QTLs for SL on chromosomes 1A, 1B, 2D, 3A, 3B, 4A, 4B, 5A, 5B, 6A, 6B, 6D, 7A, 7B, and 7D (Li et al., 2002, 2018; Marza et al., 2006; Deng et al., 2011; Cui et al., 2012a; Liu et al., 2018a; Cao et al., 2019; Chai et al., 2019; Wolde et al., 2019; Yao et al., 2019; Hu et al., 2020). It has been reported that the Q gene on chromosome 5A, which encodes an AP2 transcription factor, affects SL in wheat (Kawaura et al., 2009).

To obtain the genetic basis for HD, PH, TGW, and SL, we conducted QTL mapping based on a RIL population in the present study. In our previous study we used Bulked Segregant Analysis (BSA) and identified *VRN-B1* as the gene responsible for HD variation in the RIL population (Li et al., 2020). In this study, we used the Wheat55K SNP Array to map QTLs for HD, PH, TGW, and SL in this RIL population. Moreover, we validated the major QTLs on chromosomes 3A, 4B, and 6A by developing Kompetitive Allele Specific PCR (KASP) markers based on sequencing data and predicted candidate genes for PH, TGW, and HD according to gene annotation and SNP effects analysis.

## Materials and Methods

### Plant Materials and Phenotype Evaluation

As previously described (Li et al., 2020), a RIL population (400 lines) derived from a cross between an early heading mutant (*eh1*) and Lunxuan987 (LX987) was used for genetic mapping; generations F_6_ to F_8_ of the RIL population were included in this study. The RIL and parent lines were planted at the Zhongpuchang field station of the Institute of Crop Sciences, Chinese Academy of Agricultural Sciences (Beijing, China) during the 2015–2016, 2016–2017, and 2017–2018 cropping seasons. For each year, the experiment was conducted once and we selected three representative plants for phenotypic collection. A total of 15 plants for each line were planted in a row of 1 m, and the field conditions were managed according to local standard practices.

For HD, when more than half of the spikes had emerged from two thirds of the plants in a line, the date for that line was recorded (Li et al., 2020). At agronomic harvest maturity, three representative plants from the middle of each row showing uniform growth status were used for PH, TGW, and SL evaluation and the mean values from these three plants were used for QTL mapping. PH from each representative plant was measured from the ground to the tip of the spike excluding awns. After drying, the grain weight from each representative plant was measured and the number of grains was counted. TGW was calculated as the plant grain weight divided by the number of grains per plant multiplied by 1,000. SL from main stem of each representative plant was measured from the base of the rachis to the tip of the terminal spikelet excluding awns. The HD, PH, and TGW data were collected in 2016, 2017, and 2018, and the SL data were collected in 2016 and 2018. Analyses of variance, correlation coefficients, and broad sense heritability were performed using the ANOVA analysis tools of the QTL IciMapping v4.1 program^1^.

### Genotyping

Genomic DNA of each RIL and parent line was extracted as previously described (Li et al., 2020). After assessment of DNA integrity and quantity, the DNA from 207 lines that were also used for KASP assay, along with the parent DNA samples were hybridized to the Wheat55K SNP Array containing 53,063 markers. The genotyping was performed by China Golden Marker (Beijing) Biotech Co. Ltd^2^.

### Genetic Map Construction and QTL Analysis

High quality genotyping data were obtained by filtering with a Dish QC threshold of >0.82 and a Call-Rate threshold of >95%. The BIN function of IciMapping 4.1 was used to remove redundant markers from poly-high-resolution (PHR) SNPs, and the SNPs with >25% missing data were filtered out. The genetic map was constructed by randomly selecting only one marker from each bin using the MAP function of IciMapping 4.1. The threshold of the logarithm of odds (LOD) score was set to 2.5, and the Kosambi map function was used to calculate the map distance from recombination frequencies. Composite interval mapping (ICIM) in IciMapping 4.1 was selected to identify QTLs for HD, PH, TGW, and SL. The mean values of phenotypic traits for each line in each cropping season were used for QTL analysis. QTL region was determined by the positions of left and right markers identified by IciMapping 4.1, and physical positions of markers on the wheat reference genome v1.0 are shown in Supplementary Table 1. QTLs for the same traits identified in 2 or 3 years were considered to be stable. Multi-Environment Traits (MET) analysis of QTL IciMapping v4.1 was used for assessment of QTL × environment interactions (Li et al., 2015).

### Development of KASP Markers and QTL Validation

According to the SNPs between *eh1* and LX987 identified by RNA sequencing (RNA-seq) (Li et al., 2020), KASP markers around or in the region of stable QTLs specific for different subgenomes were designed using the polyploid primer design pipeline PolyMarker^3^. After evaluation of the polymorphisms between two parent lines, the developed KASP markers were used for genotyping the entire mapping population. The successfully developed KASP markers are listed in Supplementary Table 2. A total of 400 RILs were genotyped with KASP markers on chromosomes 3A, 4B, and 6A. The reaction volume and PCR procedures for the KASP assay were as previously described (Li et al., 2020), and the CFX 96 Real-Time System (Bio Rad, Hercules, CA, United States) was used for PCR and data analysis. QTL analysis was conducted using IciMapping 4.1.

### Analysis of SNP Effects and Prediction of Candidate Genes

Based on RNA-seq data, which was collected from young spikes of *eh1* and LX987 when *eh1* was beginning to head (Li et al., 2020), the SNPs between *eh1* and LX987 covering the intervals of flanking markers from QTL validation were obtained for SNP effects analysis. SNP effects were analyzed by Python^4^ according to the example and scripts from the website^5^. A score for missense variation is generated that reflects the predicted effect of the SNP on gene function. The more negative a score, the larger the effects on gene function. The SNPs with larger effects on gene function were speculated to be located in the candidate genes. Gene functions were predicted by searching for homologous genes in rice (*Oryza sativa*) and *Arabidopsis thaliana* using the Triticeae Multi-omics Center website^6^.

## Results

### Phenotypic Variation in the RIL Population

**FIGURE 1:**
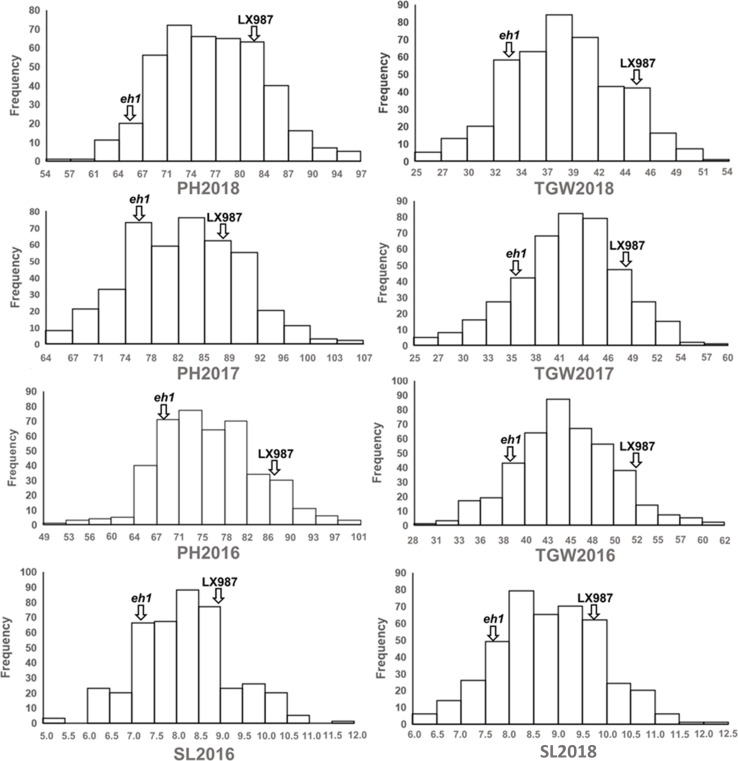
Phenotypic of distribution of plant height (PH), thousand grain weight (TGW), and spike length (SL) in the RIL population from 2016 to 2018. Phenotypic values of the two parents were marked by vertical arrows.

Our previous study showed that there is variation in HD in a RIL population of 400 lines derived from a cross between the early heading mutant *eh1* and LX987 (Li et al., 2020). In addition to HD, we also found that PH, TGW, and SL differed between *eh1* and LX987; the values of these traits were significantly lower in *eh1* than in LX987 from 2016–2018 (Table 1). Therefore, phenotypic investigation of PH, TGW, and SL in the RIL population was also conducted from 2016–2018. In the RIL population, the percent variation in PH, TGW, and SL ranged from 9.1% to 14.4% from 2016–2018, and all three traits showed moderate *h*^2^ values ranging from 0.77 to 0.82 (Table 1). In addition, PH, TGW, and SL from 2016–2018 followed a normal distribution and strong transgressive segregation was observed in the RIL population (Figure 1). Analysis of variance of PH, TGW, and SL for the multiple environment trials in the RIL population indicated that these traits were affected by environmental conditions (Supplementary Table 3).

**TABLE 1:** Summary statistics for heading date, plant height, thousand grain weight, and spike length for the two parents and the RIL population in 2016–2018.

Trait	Year	Parent		RIL population				
		*eh1*	LX987	Average	Min	Max	CV%	*h*^2^
Heading date (d)	2016	202.4	208.2	205.8	202.0	210.0	0.8	0.8
	2017	198.1	206.7	202.7	197.0	210.0	1.4	
	2018	208.8	212.3	210.6	206.0	215.0	0.7	
Plant height (cm)	2016	67.9	86.8	75.2	48.8	100.0	14.4	0.82
	2017	76.4	86.7	82.2	63.7	106.0	9.5	
	2018	66.9	80.9	76.5	54.0	95.8	9.1	
Thousand grain weight (g)	2016	39.9	52.4	44.8	28.4	60.0	11.4	0.77
	2017	36.4	48.5	41.8	24.6	58.1	13.4	
	2018	33.3	44.9	38.5	24.8	53.6	13.0	
Spike length (cm)	2016	7.5	8.6	8.1	5.0	11.7	12.8	0.79
	2018	7.7	9.2	8.5	6.0	11.3	11.2	

*Min and Max represent minimum and maximum of the corresponding traits among the RIL population. CV is coefficients of variation and h^2^ is broad sense heritability.*

Analysis of the pairwise correlations between HD, PH, TGW, and SL suggested that TGW and SL were significantly positively correlated with PH while SL was significantly negatively correlated with HD (Table 2). However, the correlations between SL and PH, SL and HD were weak (Table 2).

**TABLE 2:** Correlation coefficient analyses among heading date (HD), plant height (PH), thousand grain weight (TGW), and spike length (SL) in the RIL population.

Traits	HD	PH	TGW	SL
HD	1			
PH	0.0356^ns^	1		
TGW	0.0799^ns^	0.5570***	1	
SL	−0.2311***	0.1568**	0.0124^ns^	1

Three lines from each year were used, and the correlations are the average values of the three years. ^ns^means no significant correlation detected by statistical analysis. *** and ** indicate significance at p ≤ 0.001 and p ≤ 0.01, respectively.

### Genetic Map Construction

Among the 400 RILs, 207 lines were randomly selected for genotyping using a Wheat55K SNP Array with 53,063 tags selected from the Wheat660K SNP Array (Ren et al., 2018). Since PHR SNPs are recommended for polyploid species and have the highest reliability, only PHR SNP probes were kept. SNPs with the same genotype in both parents were removed. Finally, 6505 SNP markers were obtained for genetic map construction (Table 3). These markers were divided into 1097 unique loci with the number distributed on each chromosome ranging from 10 to 96 (Table 3). The genetic map spanned 3496.1 cM in length with an average density of 5.2 cM/locus (Table 3).

**TABLE 3:** Distribution of markers on 21 chromosomes in the constructed genetic map.

Chromosomes	No. of markers with poly high resolution	No. of unique loci	Length (cM)	cM/loci
1A	293	53	185.5	3.5
1B	164	55	110.3	2.0
1D	38	18	141.3	7.9
2A	1106	56	91.7	1.6
2B	328	80	123.5	1.5
2D	37	20	192.8	9.6
3A	392	84	243.7	2.9
3B	432	68	104.1	1.5
3D	36	14	145.2	10.4
4A	842	89	222.9	2.5
4B	277	47	86.0	1.8
4D	16	10	131.7	13.2
5A	535	95	215.9	2.3
5B	543	96	156.6	1.6
5D	50	22	264.7	12.0
6A	139	35	168.6	4.8
6B	345	49	119.2	2.4
6D	24	15	159.6	10.6
7A	458	84	254.0	3.0
7B	426	90	157.8	1.8
7D	24	17	221.3	13.0
Total	6505	1097	3496.1	5.2

### QTL Mapping Analysis

**FIGURE 2:**
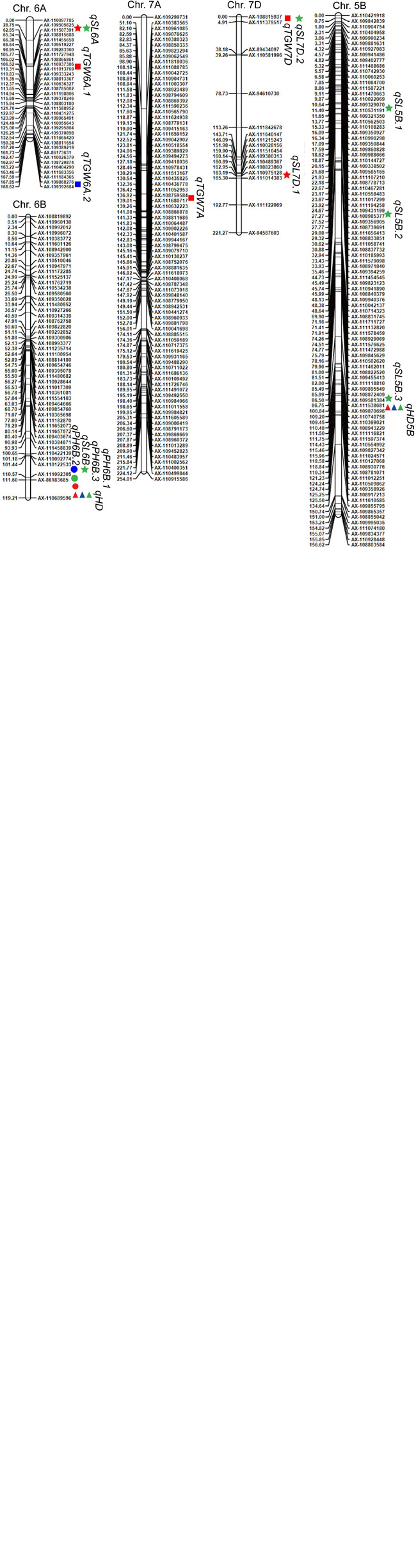
Chromosomal locations of the identified QTLs for HD, PH, TGW, and SL in 2016, 2017, and 2018. Triangles, circles, squares, and stars represent HD, PH, TGW, and SL, respectively. The colors green, blue, and red indicate data from 2016, 2017, and 2018, respectively.

A total of 37 QTLs for HD, PH, and TGW from 2016–2018, and SL from 2016 and 2018, were identified by QTL mapping analysis (Table 4 and Figure 2). These QTLs with LOD values ranging from 2.8 to 38.9 were distributed on 15 chromosomes and explained 3.0–48.8% of the phenotypic variation (Table 4 and Figure 2). There were 9, 7, 12, and 9 QTLs detected for HD, PH, TGW, and SL, respectively (Table 4 and Figure 2).

**TABLE 4:** QTLs for heading date (HD), plant height (PH), thousand grain weight (TGW), and spike length (SL) in 2016, 2017, and 2018 identified by IciMapping 4.1.

Trait and year	QTL	Position	Left marker	Right marker	LOD^a^	PVE (%)^b^	Add^c^
HD2018	*qHD2B.1*	59	AX-110960849	AX-110972149	2.8	3.6	0.3
	*qHD3A.1*	81	AX-109580196	AX-110551014	4.1	4.6	0.4
	***qHD5B***	88	AX-111538681	AX-109870696	13.6	18.4	0.7
	***qHD6B***	117	AX-86183685	AX-110689596	9.0	11.6	−0.6
HD2017	*qHD2B.2*	68	AX-110983186	AX-111716247	3.3	3.1	0.5
	*qHD3A.2*	78	AX-109103063	AX-108814864	4.3	3.5	0.6
	*qHD4A*	149	AX-108917261	AX-108763710	4.1	3.2	0.6
	***qHD5B***	88	AX-111538681	AX-109870696	38.9	48.8	2.2
	***qHD6B***	114	AX-86183685	AX-110689596	3.9	3.3	−0.6
HD2016	*qHD1B.1*	2	AX-110448009	AX-109817665	3.3	3.3	0.3
	*qHD1B.2*	110	AX-109622448	AX-109490479	5.9	6.0	0.5
	***qHD5B***	88	AX-111538681	AX-109870696	32.3	39.8	1.3
	***qHD6B***	116	AX-86183685	AX-110689596	6.8	6.4	−0.5
PH2018	*qPH2A*	65	AX-108805248	AX-110671547	3.8	6.2	1.9
	*qPH4A*	133	AX-111567358	AX-109398960	4.5	6.0	−1.8
	***qPH4B.1***	51	AX-111542943	AX-86175614	17.9	34.4	4.1
	*qPH6B.1*	118	AX-86183685	AX-110689596	3.1	4.4	1.5
PH2017	***qPH4B.1***	51	AX-111542943	AX-86175614	15.0	31.5	4.0
	*qPH6B.2*	102	AX-110122533	AX-111092305	3.0	5.6	1.7
PH2016	*qPH4B.2*	36	AX-109458638	AX-111074167	3.7	8.3	3.4
	*qPH6B.3*	111	AX-111092305	AX-86183685	4.0	8.8	3.6
TGW2018	*qTGW3B.1*	34	AX-109536560	AX-111059512	3.9	5.5	1.1
	*qTGW3D*	51	AX-110234451	AX-94739884	4.9	8.5	1.4
	***qTGW4B.1***	50	AX-109637078	AX-111542943	15.2	22.5	2.3
	*qTGW6A.1*	109	AX-110937386	AX-111013769	3.6	5.5	1.1
	*qTGW7A*	138	AX-108759584	AX-111680717	4.0	5.8	−1.2
	*qTGW7D*	0	AX-108815937	AX-111379517	4.3	6.2	1.2
TGW2017	*qTGW3B.2*	37	AX-110971226	AX-110375013	5.4	13.1	2.1
	***qTGW4B.1***	50	AX-109637078	AX-111542943	4.5	9.6	1.9
	*qTGW5D*	12	AX-111577847	AX-95658716	4.0	9.4	1.8
	*qTGW6A.2*	168	AX-109868276	AX-109392684	3.2	7.5	1.6
TGW2016	*qTGW3A*	75	AX-89583101	AX-111611367	3.6	5.8	−1.2
	*qTGW3B.3*	24	AX-109418825	AX-108890155	5.5	9.5	1.5
	*qTGW4B.2*	52	AX-108892921	AX-108871853	12.0	20.6	2.4
SL2018	*qSL4A*	126	AX-111124943	AX-108829087	3.5	3.8	0.2
	***qSL6A***	29	AX-109505625	AX-111507391	18.4	22.0	0.5
	*qSL7D.1*	165	AX-110975128	AX-111014383	5.4	6.0	−0.2
SL2016	*qSL3A*	88	AX-109584650	AX-109983808	4.2	3.0	0.2
	*qSL5B.1*	11	AX-109329070	AX-110531191	7.1	5.4	0.3
	*qSL5B.2*	25	AX-109431199	AX-108985377	14.2	11.6	−0.4
	*qSL5B.3*	86	AX-108872409	AX-109581384	11.9	9.6	−0.4
	***qSL6A***	30	AX-109505625	AX-111507391	11.1	9.5	0.4
	*qSL6B*	110	AX-110122533	AX-111092305	12.4	10.1	0.4
	*qSL7D.2*	1	AX-108815937	AX-111379517	6.7	5.1	−0.3

*The QTLs written in bold font were detected in more than 1 year. ^a^LOD values of detected QTL. ^b^Phenotypic variation explained by the corresponding QTL. ^c^Additive effect of the corresponding QTL. Positive values indicate the alleles from LX987 increase the trait values, and negative values indicate the alleles from eh1 increase the corresponding trait values.*

QTLs for HD were detected on chromosomes 1B (2), 2B (2), 3A (2), 4A (1), 5B (1), and 6B (1) (Table 4 and Figure 2). Notably, *qHD5B* and *qHD6B* were detected in all 3 years. *qHD5B* explained 18.4–48.8% of the phenotypic variation while *qHD6B* accounted for 3.3–11.6% of the phenotypic variation (Table 4). *qHD3A.1* was located close to *qHD3A.2*, and *qHD2B.1* was located close to *qHD2B.2*. *qHD2B.1* was detected in 2018 and explained 3.6% of the phenotypic variation, and *qHD2B.2* was detected in 2017 and explained 3.1% of the phenotypic variation (Table 4). For all of the QTLs except *qHD6B* the allele increasing HD was contributed by LX987 (Table 4).

For PH, 7 QTLs were identified on chromosomes 2A (1), 4A (1), 4B (2), and 6B (3) (Table 4 and Figure 2). *qPH4B.1* was identified in 2017 and 2018 (Table 4 and Figure 2), with LOD scores of 15.0 and 17.9 and explaining 31.5% and 34.4% of the phenotypic variation, respectively (Table 4). Three co-located QTLs were identified on chromosome 6B from 2016–2018, which explained 4.4%–8.8% of the variation in PH (Table 4 and Figure 2). For all of the QTLs except *qPH4A* the allele increasing PH was contributed by LX987 (Table 4).

For TGW, 12 QTLs were detected on chromosomes 3A (1), 3B (3), 3D (1), 4B (2), 5D (1), 6A (2), 7A (1), and 7D (1) (Table 4 and Figure 2). *qTGW4B.1* was detected in 2017 and 2018, explaining 9.6% and 22.5% of the variation in TGW, respectively (Table 4). *qTGW4B.2*, which was located close to *qTGW4B.1*, was detected in 2016 and explained 20.6% of the phenotypic variation (Table 4). In addition, the QTLs *qTGW3B.1* and *qTGW3B.2* were located close to each other and explained 5.5% and 13.1% of phenotypic variation in 2018 and 2017, respectively (Table 4). For all of the QTLs except *qTGW7A* and *qTGW3A*, the allele increasing TGW was contributed by LX987 (Table 4).

For SL, 9 QTLs were identified on chromosomes 3A (1), 4A (1), 5B (3), 6A (1), 6B (1), and 7D (2). *qSL6A* was detected in 2016 and 2018, explaining 9.5% and 22.0% of phenotypic variation, respectively (Table 4). *qSL5B.2* and *qSL5B.3* showed high contributions to phenotypic variation, 11.6% and 9.6%, respectively (Table 4). For *qSL7D.1*, *qSL5B.2*, *qSL5B.3*, and *qSL7D.2*, the negative alleles were contributed by LX987 while for the other QTLs the allele increasing SL was contributed by LX987 (Table 4).

Four QTL clusters were identified on chromosomes 3A, 4B, 5B, and 6B (Table 5). For the QTL cluster on chromosome 3A, *qHD3A.1* and *qHD3A.2* were co-localized with *qTGW3A* and *qSL3A* in a region ranging from 70.28 cM to 88.01 cM. On chromosome 4B, *qPH4B.1* for PH was clustered with two QTLs for TGW, with the alleles from LX987 increasing PH and TGW. For the QTL cluster on chromosome 5B, *qHD5B*, which was detected in all 3 years, was clustered with *qSL5B.3* (Tables 4, 5); however, the positive alleles for these QTLs were derived from opposite parents (Table 4). Three QTLs for PH on chromosome 6B were clustered with *qHD6B* and *qSL6B*, with the alleles from LX987 increasing PH and SL (Tables 4, 5).

**TABLE 5:** QTL clusters affecting two or more traits. QTLs from each year located within 10 cM and affected two or more traits were identified as a QTL cluster.

Chromosome	QTL	Marker interval	Position (cM)
3A	*qHD3A.1*, *qHD3A.2*, *qTGW3A*, *qSL3A*	AX-89583101–AX-109983808	70.28–88.01
4B	*qPH4B.1*, *qTGW4B.1*, *qTGW4B.2*	AX-109637078–AX-108871853	49.40–52.19
5B	*qHD5B*, *qSL5B.3*	AX-108872409–AX-109870696	85.99–100.84
6B	*qHD6B*, *qPH6B.1*, *qPH6B.2*, *qPH6B.3*, *qSL6B*	AX-110122533–AX-110689596	101.44–119.21

To evaluate the QTL × environment interactions, Multi-Environment Traits (MET) analysis was employed by using QTL IciMapping v4.1 (Li et al., 2015). Similarly, 33 QTLs were identified by MET analysis (Supplementary Table 4). Among them, 10 QTLs showed significant interactions with environment, including the major QTLs *qHD5B*, *qPH4B.1*, and *qTGW4B.1*.

### QTL Validation by Mapping With Molecular Markers

**FIGURE 3:**
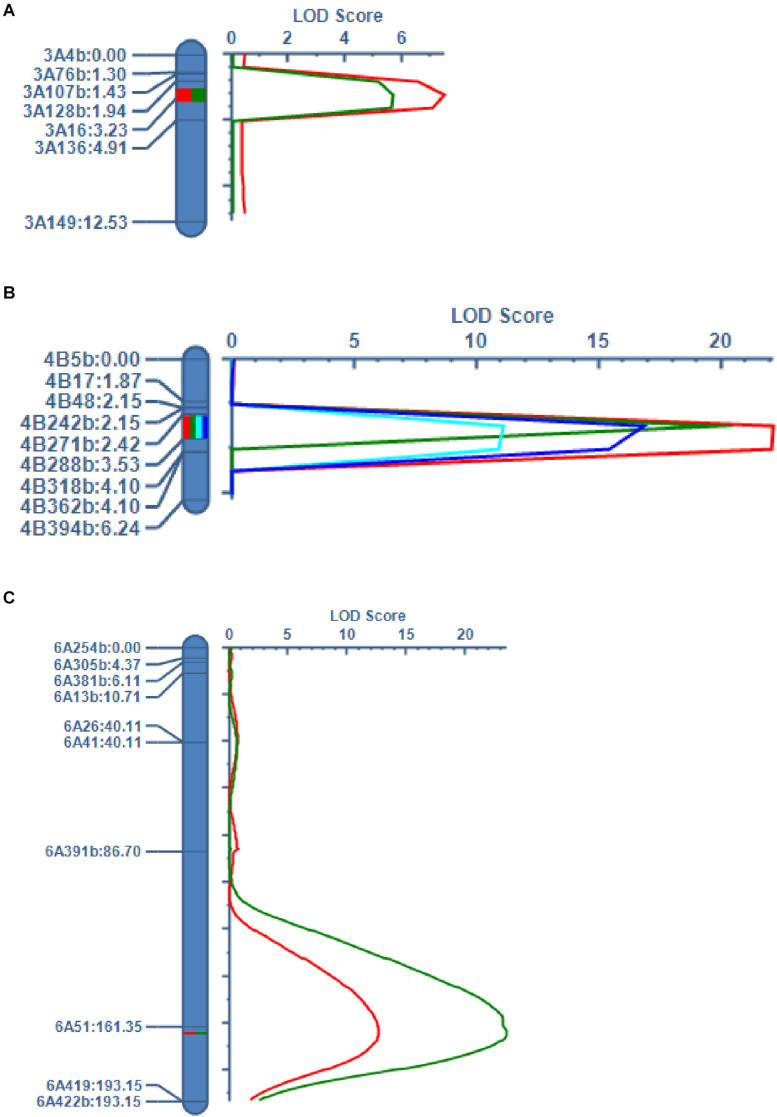
QTL validation using KASP markers developed based on RNA-seq data. **(A)** QTLs for HD on chromosome 3A. The green and red curves represent LOD scores from 2017 and 2018, respectively. **(B)** QTLs for PH and TGW on chromosome 4B. The red and green curves represent LOD scores for PH from 2017 and 2018, respectively. The light blue and blue curves represent LOD scores for TGW from 2016 and 2018, respectively. **(C)** QTLs for SL on chromosome 6A. The red and green curves represent LOD scores from 2016 and 2018, respectively. The marker name and genetic position of each marker are indicated on the left side of each chromosome.

The QTLs on chromosomes 3A (*qHD3A*), 4B (*qPH4B.1* and *qTGW4B.1*), 5B (*qHD5B*), and 6A (*qSL6A*) were stably detected in different years. We selected these QTLs for validation using KASP markers developed based on RNA-seq data (Li et al., 2020). In a recent study we reported that the *VRN-B1* gene located on chromosome 5B around the *qHD5B* region was responsible for HD variation in the RIL population (Li et al., 2020). For the validation of *qHD3A*, we successfully developed seven KASP markers around or in the 55K SNP array-mapped region, and delimited the QTL to a genetic interval of 1.29 cM between markers 3A128b and 3A16, spanning approximately 2.5 Mb (Figure 3A). The LOD scores of this QTL were 5.7 and 7.5, explaining 6.0% and 8.0% of the variation of HD, in 2017 and 2018, respectively (Table 6). For the QTLs on chromosome 4B, nine KASP markers were successfully developed, and the QTLs for PH were narrowed down to a genetic interval of 1.11 cM flanked by markers 4B271b and 4B288b, corresponding to an approximately 0.8 Mb physical region (Figure 3B). This QTL was detected in 2017 and 2018, with LOD scores of 22.1 and 19.8, and explaining 22.8% and 20.5% of the variation in PH, respectively (Table 6). Consistent with this, the QTL for TGW identified in 2016 and 2018 was mapped between markers 4B271b and 4B288b with LOD scores of 11.1 and 16.8 and explaining 12.2% and 17.8% of the variation in TGW, respectively (Figure 3B and Table 6). For the validation of *qSL6A*, we successfully developed 10 KASP markers for genetic mapping. In 2016 and 2018, a major QTL for SL with LOD scores of 12.6 and 23.5 and explaining 13.1% and 22.5% of phenotypic variation, respectively, was detected between markers 6A51 and 6A419 within a genetic interval of 31.8 cM (Figure 3C and Table 6). Due to the large region for this QTL, we did not conduct further analysis of the candidate genes.

**TABLE 6:** Details of the genetic map for QTL validation generated using KASP markers.

Trait name	Chromosome	Position	Left marker	Right marker	LOD	PVE (%)	Add
HD2018	3A	3	3A128b	3A16	7.5	8.0	0.4
HD2017	3A	3	3A128b	3A16	5.7	6.0	0.8
PH2017	4B	3	4B271b	4B288b	22.1	22.8	3.7
PH2018	4B	3	4B271b	4B288b	19.8	20.5	3.2
TGW2016	4B	3	4B271b	4B288b	11.1	12.2	1.8
TGW2018	4B	3	4B271b	4B288b	16.8	17.8	2.1
SL2016	6A	164	6A51	6A419	12.6	13.1	0.4
SL2018	6A	164	6A51	6A419	23.5	22.5	0.5

*The 400 RILs were used for QTL validation.*

### Gene Annotations and Effects of SNPS in the Validated QTL Regions on Chromosome 4B and 3A

Since *qPH4B.1* and *qTGW4B.1* were delimited to a physical interval of 0.8 Mb between markers 4B271b and 4B288b by genetic mapping with KASP markers, we analyzed gene models and annotations in this region according to the Chinese Spring (CS) reference genome v1.0 (International Wheat Genome Sequencing Consortium, 2018). In this region, seven high-confidence genes were annotated. Based on BLASTP searches for rice and *Arabidopsis* homologous genes^7^, these genes are predicted to encode 40S ribosomal protein S27 (TraesCS4B01G280800), Beta-galactosidase (TraesCS4B01G280900), a Histidine-containing phosphotransfer protein (TraesCS4B01G281000), 60S ribosomal protein L5 (TraesCS4B01G281100), Protein PAM68 (TraesCS4B01G281200), Tribbles homolog 3 (TraesCS4B01G281300), and a Ubiquitin carboxyl-terminal hydrolase family protein (TraesCS4B01G281400) (Supplementary Table 5). We also analyzed sequence variation between LX987 and *eh1* in this region based on RNA-seq data. A total of 18 SNPs with each parent homozygous for different alleles were identified (Supplementary Table 6). Analysis of SNP effects suggested that three SNPs were missense mutations. One SNP in TraesCS4B02G281200 located at the 189^*th*^ position caused a change in the amino acid Leu in LX987 to Trp in *eh1* and was predicted to have the largest effect on gene function. Multiple alignment of amino acid sequences of protein PAM68 from grasses indicated that this region is conserved among *Brachypodium distachyon*, *Sorghum bicolor*, *Zea mays* and rice (Supplementary Figure 2).

For the HD QTL on chromosome 3A between markers 3A128b and 3A16, we found that 38 high-confidence genes were annotated in the mapped interval (Supplementary Table 7). In this region, nine homozygous SNPs with genotypes differing between the two parent lines were found based on RNA-seq data. One SNP in TraesCS3A01G086400, which encodes an R3H domain-containing protein, that caused a change from Ser to Pro at the 267^th^ position had the largest effect on gene function (Supplementary Table 8).

## Discussion

### QTL Mapping Using the WHEAT55K SNP Array

SNP arrays are a powerful and effective approach for QTL mapping (Rasheed et al., 2017). The tags of the Wheat55K Array (Affymetrix^®^ Axiom^®^ Wheat55) were carefully selected from the Wheat660K Array, and all tags were uniformly distributed on 21 chromosomes. Therefore, the 55K Array is suitable for genotyping in QTL studies (Ren et al., 2018). The Wheat55K SNP Array has been utilized for QTL mapping of productive tiller number (Liu et al., 2018b), temporal expression of tiller number (Ren et al., 2018), and leaf rust and stripe rust resistance (Huang et al., 2019; Zhang et al., 2019) in wheat. In this study, we used the Wheat55K SNP Array to genotype 207 RILs and constructed a genetic map containing 6,505 PHR SNP markers (Table 3). PHR SNPs are of high quality and possess better cluster resolution than other SNPs (Marrano et al., 2019), which improves the accuracy of genotyping. The genetic map spanned 3496.1 cM across the 21 chromosomes, which is similar to the total length of genetic maps for 199 wheat RILs constructed by Liu et al. (2018b) and 186 RILs constructed by Huang et al. (2019). We detected a total of 37 QTLs for HD, PH, TGW, and SL by mapping using the 55K SNP array (Table 4 and Figure 2). Among these QTLs, those on chromosomes 3A (*qHD3A*), 4B (*qPH4B.1* and *qTGW4B.1*), 5B (*qHD5B*) (Li et al., 2020), and 6A (*qSL6A*) that were stably detected in different years, were validated using KASP markers (Figure 3). High LOD values ranging from 5.7 to 23.5 were observed for the QTLs that were validated with KASP markers (Table 6), indicating that the QTLs detected using the 55K SNP array data are reliable.

### Comparison of the Mapped QTLS With Those Identified in Previous Studies

A total of nine QTLs for HD were mapped on chromosomes 1B, 2B, 3A, 4A, 5B, and 6B (Table 4 and Figure 2). Consistent with these findings, in our previous study we also identified QTLs for HD on chromosomes 2B, 3A, and 5B using BSA of the same RIL population (Li et al., 2020). In addition, *qHD1B.1* and *qHD4A* were mapped to genetic regions similar to those reported by Zhao et al. (2019). *qHD2B.2* was mapped to a genetic position similar to that of HD QTLs reported by Hu et al. (2020) and Li et al. (2018). The analysis of the physical positions of the flanking markers in the wheat reference genome indicated that *qHD2B.2* is probably the *Ppd-B1* gene. The two adjacent QTLs *qHD3A.1* and *qHD3A.2* are located at a genetic position similar to that of an HD QTL reported by Li et al. (2018). The QTL *qHD5B*, which was stably detected in different years (Table 4 and Figure 2), is located around gene *VRN-B1*, and our previous results suggested that the *VRN-B1* gene is responsible for HD variation in this RIL population (Li et al., 2020). In addition, the stably detected QTL *qHD6B* was found at a position similar to that of HD QTLs reported by Perez-Lara et al. (2016) and Li et al. (2018).

Regarding PH, we identified two and three QTLs on chromosomes 4B and 6B, respectively (Table 4 and Figure 2). Consistent with these results, previous studies also reported several PH QTLs on chromosomes 4B and 6B (Gao et al., 2016; Li et al., 2018; Jahani et al., 2019). *qPH4B.2*, *qPH6B.1*, *qPH6B.2*, and *qPH6B.3* were mapped to similar genetic positions on chromosomes 4B and 6B as PH QTLs reported by Gao et al. (2015); Li et al. (2018), and Hu et al. (2020). BLAST searches of flanking markers for *qPH4B.2* against the wheat reference genome indicated that this region harbors the reported *RhtB1b* gene (Peng et al., 1999). However, using KASP markers for *Rht1* (Rasheed et al., 2016) we found that both of the parent lines, *eh1* and LX987, had the same *RhtB1b* genotype (Supplementary Figure 1), indicating that the detection of *qPH4B.2* is not due to *RhtB1b*. It is possible that there is other variation in the *Rht1* gene that causes differences in PH between the parent lines. *qPH2A* is located at a similar genetic position as a QTL reported by Li et al. (2018), and the *qPH4A* region overlaps with the physical region reported by Chen et al. (2020). It has been reported that the semi-dominant dwarfing gene *Rht-NM9* is located in a region from 178.9 Mb to 187.2 Mb on 2AS (Lu et al., 2015). We performed a BLAST search using the flanking markers for *qPH2A* and found that this QTL is located in a 143–148 Mb interval according to the CS reference genome. Therefore, we speculate that *qPH2A* does not harbor the *Rht-NM9* gene.

The stably detected QTL *qTGW4B.1* co-localized with *qTGW4B.2* (Table 4 and Figure 2). These QTLs are located within 49.4–52.19 cM. Guan et al. (2018) reported stable QTLs for TGW located within 22.3–95.8 cM on chromosome 4B. *qTGW3A* is located at a genetic position similar to that reported in Cui et al. (2014a). *qTGW3D* is located at positions similar to those reported in Cui et al. (2014b) and Gao et al. (2015). The QTLs *qTGW3B.1*, *qTGW3B.2*, *qTGW3B.3*, *qTGW5D*, and *qTGW7A* are located at positions similar to those reported by Li et al. (2018), and *qTGW6A.1* is located close to a stable yield and TGW QTL reported by Simmonds et al. (2014). In addition, the QTLs *qTGW7A* and *qTGW7D* are located at similar genetic positions as those reported by Cui et al. (2014a) and Guan et al. (2018), respectively.

*qSL4A* is located at a genetic position similar to that reported by Cui et al. (2012b) and Gao et al. (2015). *qSL5B.2* is located at a position similar to that in Cui et al. (2012b). In addition, *qSL6B* is located at a genetic position similar to that reported by Li et al. (2018) and Hu et al. (2020). To the best of our knowledge, the stably detected QTL *qSL6A* with a LOD value ranging from 11.1 to 18.4 is likely to be a new QTL (Table 4 and Figure 2).

### Pleiotropic QTLS for HD, PH, TGW, and SL

Among the QTLs for HD, PH, TGW, and SL detected in this study, four regions controlled two or more of these traits (Table 5). In addition to *Rht1*, a previous study identified a “QTL-hotspot” region for yield-related traits on chromosome 4B (Guan et al., 2018). This is consistent with the QTL cluster detected in our study (Table 5). Consistent with the positive correlation between PH and TGW (Table 2 and Supplementary Table 9), the superior alleles of the co-localized QTLs *qPH4B.1*, *qTGW4B.1*, and *qTGW4B.2* were derived from the same parent line (Table 4). A QTL cluster for HD, PH, and SL that mapped to the interval 101.44–119.21 cM on chromosome 6B (Table 5) is likely the same or similar to a QTL cluster for yield-related traits reported by Li et al. (2018). *qHD6B* co-localized with *qSL6B*, with favorable alleles derived from opposite parents (Tables 4, 5). This is consistent with the negative correlation between HD and SL (Table 2).

### Candidate Genes Affecting PH, TGW, and HD

Using KASP markers, we delimited the QTL regions for PH and TGW on chromosome 4B to a 0.8 Mb physical region (Figure 3B and Table 6). A recent study identified a QTL cluster for TGW linked to *Rht-B1* on chromosome 4B using near-isogenic lines (Guan et al., 2020). This region includes the physical interval identified in our study. According to gene annotation and analysis of the effects of SNPs in the mapped region (Supplementary Table 6), a mutation in TraesCS4B02G281200 encoding a PAM68 protein showed the largest effect on gene function. The PAM68 protein is essential for efficient D1 biogenesis and photosystem II assembly in *Arabidopsis* (Armbruster et al., 2010, 2013). Split-ubiquitin assays suggested that the C terminus of *Arabidopsis* PAM68 is required for interaction with the PSII core proteins D1 and CP43 (Armbruster et al., 2010). The variation in the PAM68 protein between LX987 and *eh1* is located at the C terminus, and this region is conserved in grasses (Supplementary Figure 2). This indicates that the mutation in PAM68 may affect gene function. Photosynthesis plays an important role in yield improvement (Zhu et al., 2010). It has been reported that mutation of the photosystem 1-F subunit (*OsPS1-F*) results in reduction of PH and grain yield in rice (Ramamoorthy et al., 2018). Taken together, our results and previous findings suggest that PAM68 is a candidate gene for the PH and TGW QTLs. We found genes TraesCS4B01G281000 and TraesCS4B01G281300 in the QTL region were not expressed in the RNA-seq data, which may due to that the RNA-seq data was collected from spikes in the HD (Li et al., 2020). Therefore, we could not exclude these two genes as candidate genes from the sequences of RNA and the predicted effects of SNP. However, TraesCS4B01G281000 and TraesCS4B01G281300 encode Histidine-containing phosphotransfer protein and Tribbles homolog 3, respectively, which have not been reported for involving in PH and TGW. In this respect, the possibility for these two candidate genes is low.

We also confirmed and narrowed down the QTL region for HD on chromosome 3A to a 2.5 Mb interval (Figure 3A and Table 6). Analysis of SNP effects suggested that a mutation in TraesCS3A01G086400 has a large effect on gene function (Supplementary Table 8), suggesting that this gene may affect HD in the RIL population. TraesCS3A01G086400 encodes an R3H domain-containing protein, which functions in binding polynucleotides, including DNA, RNA, and single-stranded DNA (Grishin, 1998). Studies on R3H-containing proteins in maize (Saleh et al., 2006) and *Arabidopsis* (Wang et al., 2019) have suggested that these proteins are involved in stress responses. Whether the R3H domain-containing protein contributes to HD variation need to be further studied.

## Conclusion

We identified 37 QTLs for HD, PH, TGW, and SL in a RIL population using the Wheat55K SNP Array, and validated the stably detected QTLs on chromosome 3A, 4B, and 6A using KASP markers. The QTLs on chromosomes 4B and 3A were delimited to a physical interval of 0.8 Mb and 2.5 Mb, respectively. Moreover, the candidate genes affecting PH, TGW, and HD were predicted based on gene annotation and analysis of SNP effects. The linked KASP markers developed in this study will facilitate breeding for yield improvement in wheat.

## Data Availability Statement

The original contributions presented in the study are included in the article/Supplementary Material, further inquiries can be directed to the corresponding author/s.

## Author Contributions

LL conceived the project and revised the manuscript. HX and YL conducted most of the experiments. HG constructed the RIL population and assisted in collection of the phenotypic data. YX and JG performed genotype analyses. LZ, SZ, and YD participated in field trials. HX wrote the first draft of the manuscript. All authors have read and approved the final manuscript.

## Conflict of Interest

The authors declare that the research was conducted in the absence of any commercial or financial relationships that could be construed as a potential conflict of interest.

## Footnotes

1 http://www.isbreeding.net/

2 http://www.cgmb.com.cn/

3 http://polymarker.tgac.ac.uk/

4 https://www.python.org/

5 https://github.com/pinbo/gene_manual_annotation

6 http://202.194.139.32/searchtools/

7 http://202.194.139.32/searchtools/

## Supplementary Material

Supplementary Figure 1 Genotypes of *eh1* and LX987 obtained using the *Rht1* KASP markers.

Supplementary Figure 2 Multiple sequence alignment of PAM68 in *Brachypodium distachyon*, barley, rice, sorghum, maize, and wheat. The labeling with red asterisk indicates the position of variation between the two parent lines.

Supplementary Table 1 Physical positions of left and right markers of identified QTLs.

Supplementary Table 2 The primers used for KASP assays.

Supplementary Table 3 Variance analysis of PH, TGW, and SL in the 400 RIL. ^∗∗∗^ indicates significance at *p* ≤ 0.001.

Supplementary Table 4 MET analysis of QTLs by environment interactions. QTL with a LOD (AbyE) >2.5 was significant interaction with environment.

Supplementary Table 5 High-confidence genes located between markers 4B271b and 4B288b.

Supplementary Table 6 SNPs identified using RNA-seq data for LX987 and *eh1* in the mapped region of chromosome 4B.

Supplementary Table 7 High-confidence genes located between markers 3A128b and 3A16.

Supplementary Table 8 SNPs identified using RNA-seq data for LX987 and *eh1* in the mapped region of chromosome 3A.

Supplementary Table 9 Correlation coefficient analyses of HD, PH, TGW, and SL among different years.
