# Supplementary figures and images for: Genetic Mapping by Integration of 55K SNP Array and KASP Markers Reveals Candidate Genes for Important Agronomic Traits in Hexaploid Wheat
Source: Front Plant Sci. 2021 Feb 23;12:628478. doi: 10.3389/fpls.2021.628478 (PMC7942297; doi:10.3389/fpls.2021.628478)

## Slide 1
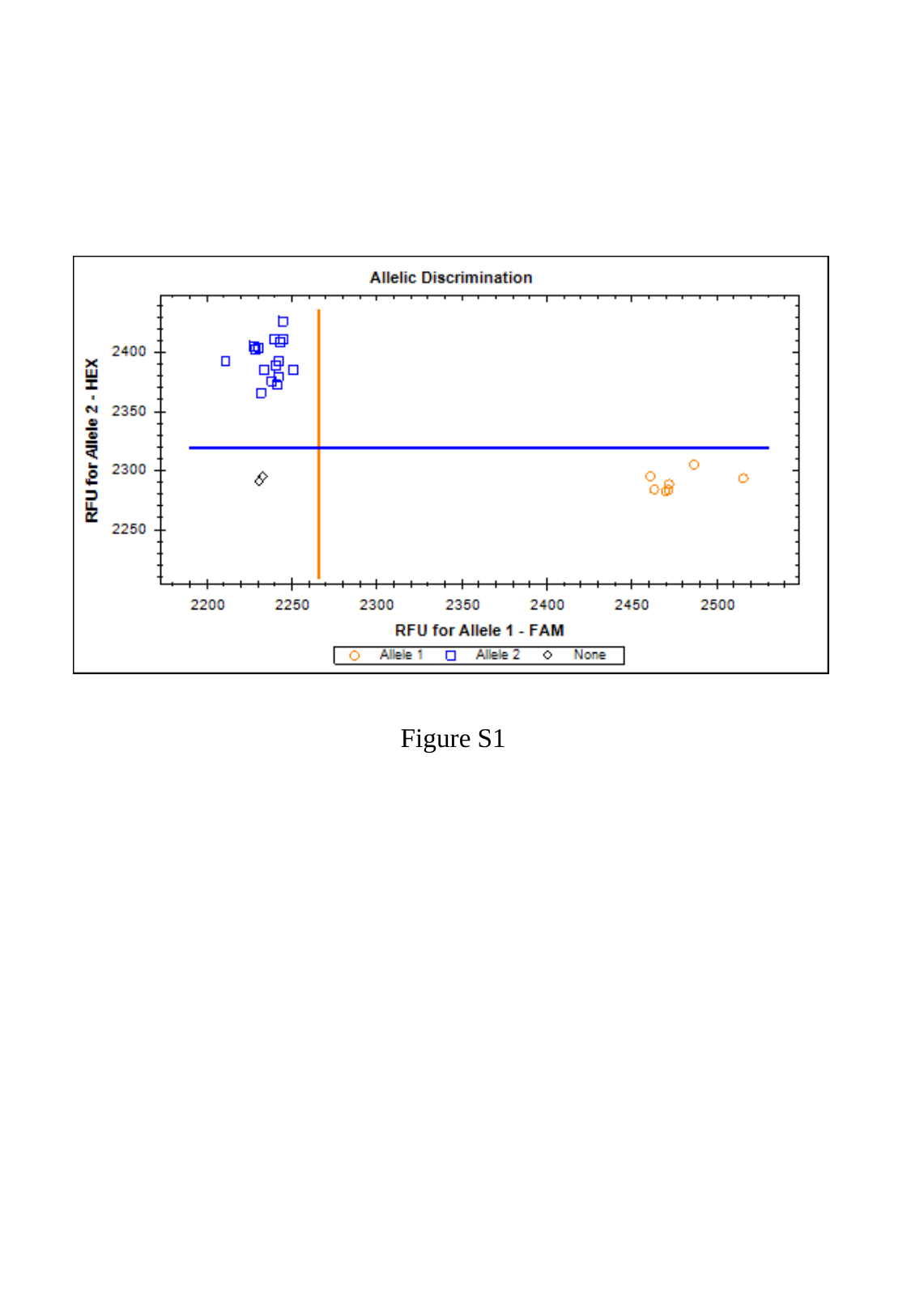

Figure S1

## Slide 2
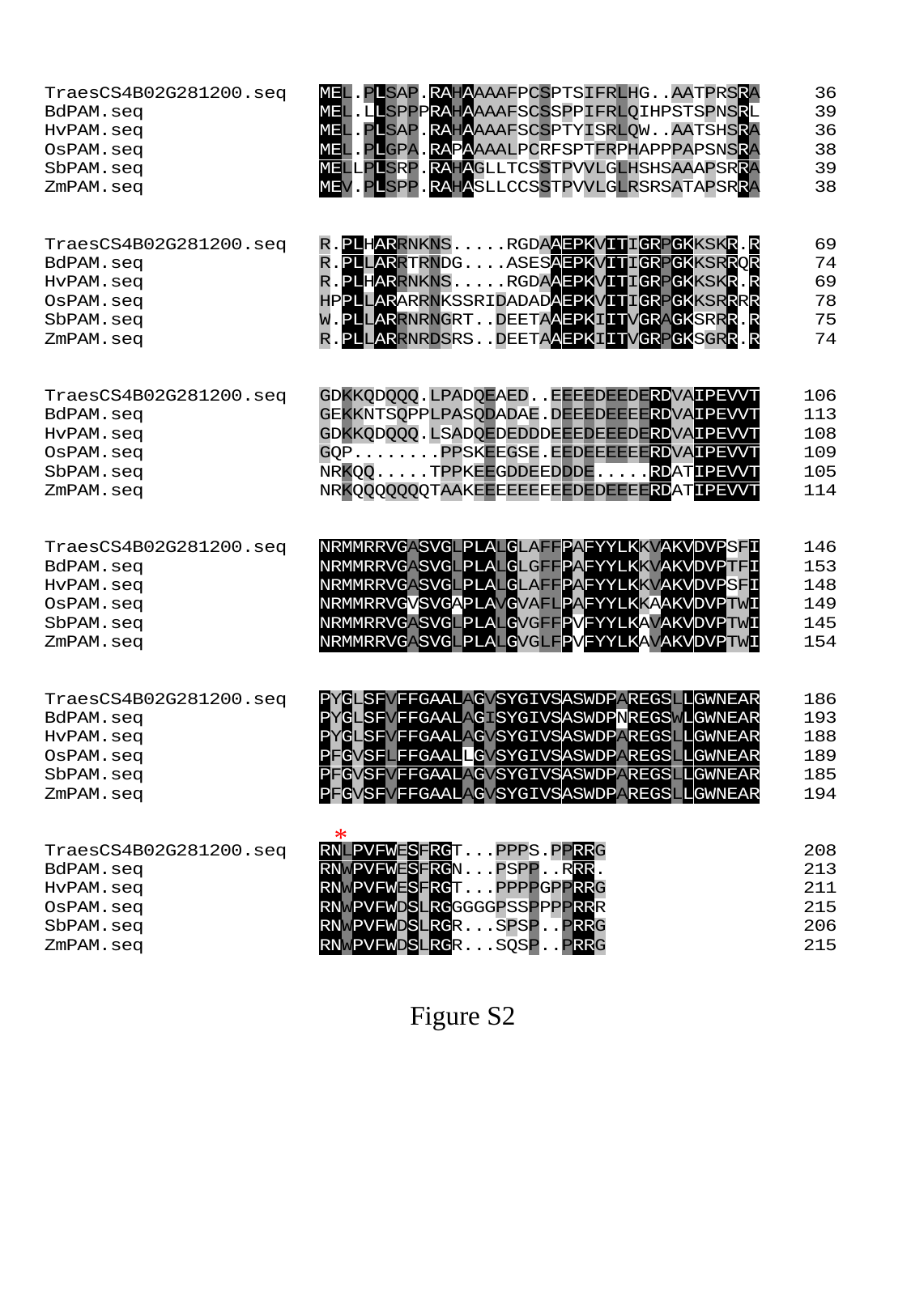

*
Figure S2

Supplement: Supplementary Figure 1 — Genotypes of eh1 and LX987 obtained using the Rht1 KASP markers. [file Presentation_1.PPTX]
